# Hemodynamic consequences of severe lactic acidosis in shock states: from bench to bedside

**DOI:** 10.1186/s13054-015-0896-7

**Published:** 2015-04-09

**Authors:** Antoine Kimmoun, Emmanuel Novy, Thomas Auchet, Nicolas Ducrocq, Bruno Levy

**Affiliations:** CHU Nancy, Service de Réanimation Médicale Brabois, Pole Cardiovasculaire et Réanimation Médicale, Hôpital de Brabois, Vandoeuvre-les-Nancy, 54511 France; Université de Lorraine, Nancy, 54000 France; INSERM U1116, Groupe Choc, Faculté de Médecine, Vandoeuvre-les-Nancy, 54511 France

## Abstract

Lactic acidosis is a very common biological issue for shock patients. Experimental data clearly demonstrate that metabolic acidosis, including lactic acidosis, participates in the reduction of cardiac contractility and in the vascular hyporesponsiveness to vasopressors through various mechanisms. However, the contributions of each mechanism responsible for these deleterious effects have not been fully determined and their respective consequences on organ failure are still poorly defined, particularly in humans. Despite some convincing experimental data, no clinical trial has established the level at which pH becomes deleterious for hemodynamics. Consequently, the essential treatment for lactic acidosis in shock patients is to correct the cause. It is unknown, however, whether symptomatic pH correction is beneficial in shock patients. The latest Surviving Sepsis Campaign guidelines recommend against the use of buffer therapy with pH ≥7.15 and issue no recommendation for pH levels <7.15. Furthermore, based on strong experimental and clinical evidence, sodium bicarbonate infusion alone is not recommended for restoring pH. Indeed, bicarbonate induces carbon dioxide generation and hypocalcemia, both cardiovascular depressant factors. This review addresses the principal hemodynamic consequences of shock-associated lactic acidosis. Despite the lack of formal evidence, this review also highlights the various adapted supportive therapy options that could be putatively added to causal treatment in attempting to reverse the hemodynamic consequences of shock-associated lactic acidosis.

## Introduction

Shock was recently redefined as a clinical state of acute circulatory failure with inadequate oxygen utilization and/or delivery by the cells resulting in cellular dysoxia/hypoxia [1]. In this setting, shock-associated lactic acidosis is the principal but not exclusive cause of metabolic acidosis in the shock state. Current clinical practice considers a pH ≤7.35 and lactatemia >2.0 mmol.l^−1^ with a PaCO_2_ ≤ 42 mmHg as defining lactic acidosis [2,3]. In contrast, the definition of severe lactic acidosis is unclear. Critical care physicians usually consider that metabolic acidosis with a pH <7.2 has deleterious hemodynamic effects and requires symptomatic treatment [4]. Nevertheless, despite optimal management with adequate supportive and etiological therapy, shock and severe lactic acidosis (that is, with pH <7.2) remain associated with an observed high mortality rate of about 50%, while no survival has been reported for severe lactic acidosis with shock under pH 7.0 [5-8].

Numerous studies have assessed the cardiovascular consequences of severe metabolic acidosis, including lactic acidosis. These experimental studies demonstrated that severe metabolic acidosis worsens cardiovascular function [9,10] by exacerbating myocardial dysfunction and hyporesponsiveness to vasopressors [11]. Nevertheless, such findings have yet to be formally observed in human studies.

Etiological treatment is essential while symptomatic lactic acidosis correction remains a contentious issue. It is unknown whether alkalinization is beneficial in severe lactic acidosis. The Surviving Sepsis Campaign recommends against symptomatic treatment in lactic acidotic patients with a pH >7.15 for the purpose of improving hemodynamic status [2]. Alternatively, the effect of alkalinization on hemodynamics and vasopressor requirements at pH ≤7.15 is currently unknown. Nevertheless, despite the lack of relevant results on its efficacy, alkalinization is still largely prescribed in instances of severe acidosis with pH ≤7.15 [4].

The present review was written based on a critical and personal appraisal of the literature from 1 January 1980 to 1 December 2014 searched for using the MEDLINE database. The object of the search was the hemodynamic consequences of lactic acidosis during the shock state. The following terms were searched and combined: ‘bicarbonate’ , ‘metabolic acidosis’ , ‘lactic acidosis’ , ‘pH’ , ’shock’ , ‘renal replacement therapy’ , and ‘anion gap acidosis’. In addition, references from each identified article were carefully reviewed for additional suitable references. Studies involving humans or animals were examined, and the search was restricted to articles published in the English language.

This review focuses only on the hemodynamic consequences of severe lactic acidosis with appropriate response of the ventilatory system; that is, pH <7.2, PCO_2_ ≤ 42 mmHg and lactatemia >5 mmol.l^−1^ (approximation based on the Henderson-Hasselbach equation for PCO_2_ = 42 mmHg). The complex association of respiratory and metabolic acidosis is not discussed in this article. Symptomatic therapeutic options are also reviewed. However, other types of metabolic acidosis, such as isolated acute renal failure, bicarbonate-losing metabolic acidosis, ketoacidosis, hyperchloremic acidosis or metformin-induced lactic acidosis, are not addressed, aside from specific comparisons.

## Epidemiology and outcome of severe lactic acidosis

Lactic acidosis is one of the most common biological concerns for intensivists. Nevertheless, clinical studies assessing the incidence and outcome of lactic acidosis are sparse and are mostly retrospective or prospective in nature with small sample sizes.

For this review, the most convincing prospective multi-center study, conducted in 2011 by Jung and colleagues [12], noted severe lactic acidosis in 6% of the studied population (200/2,550 patients); that is, with pH 7.09 ± 0.11 with high lactatemia values. Eighty-three percent of these patients were treated with vasopressors with a mortality rate of 57%. In this study, lactatemia and the swiftness of lactic acidosis correction were linked with survival. Interestingly, only 18% exhibited a slight coexistent respiratory acidosis at admission.

Clearly, a causal relationship between lactic acidosis and mortality has yet to be established. For example, in metformin-associated lactic acidosis, even with pH values most often around 7.0, the observed mortality rate was 25% [13]. However, for the same pH values during shock, regardless of origin, no survival was reported [8]. Consequently, severe lactic acidosis is much more of a precipitator than a direct causal factor of mortality. Lactic acidosis probably contributes to the decompensation of underlying comorbidities and, hence, to the mortality rate.

## Lactate generation in shock states

As indicated above, lactic acidosis is a common phenomenon in shock patients and a high predictor of mortality. The pathophysiology of shock-associated lactic acidosis is still taught to medical students as a direct marker of oxygen debt or hypoperfusion in tissues (type A lactic acidosis) [14]. Lactate is produced from pyruvate and through the glycolysis cascade. Thus, when pyruvate production exceeds mitochondrial capacity, lactate generation increases.

Far from being the only hypothesis explaining hypoxia-induced hyperlactatemia, numerous other mechanisms are involved, including under aerobic conditions (type B lactic acidosis). Indeed, lactate is first and foremost an energetic, non-toxic substratum. Under resting conditions, half of the total lactate produced (1,500 mmol.day^−1^) is directed toward gluconeogenesis in the liver (Cori cycle) while the remaining 50% is consumed via oxidation [15]. Moreover, the kidney is also involved, acting as a neoglucogenesis-directed metabolizer in the cortex and as a producer of lactate in the medulla. At the cellular level, in response to adrenergic stress in shock patients, accelerated glycolysis enhances lactate production [16]. An elevated lactate/pyruvate ratio is an indicator of a cytoplasmic accumulation of NADH that can be used to regenerate ATP [17]:

$$ \mathrm{A}\mathrm{D}\mathrm{P} + \mathrm{NA}\mathrm{D}\mathrm{H} + {\mathrm{H}}^{+}= > \mathrm{A}\mathrm{T}\mathrm{P} + \mathrm{N}\mathrm{A}\mathrm{D} $$

Thus, the increase in lactate/pyruvate ratio appears to be much more of an adaptive response to shock-induced energetic debt than an actual side effect [18].

In shock patients, acute liver or renal dysfunctions are most often associated with decreased lactate clearance and a pronounced increase in blood lactate level compared with patients without liver or renal dysfunction. However, liver and renal dysfunctions are inextricably linked with the shock state and their impact on the decreased lactate clearance in this situation remains unclear [19,20].

## Is hyperlactatemia systematically associated with metabolic acidosis?

At first glance, it might appear somewhat counterintuitive that lactate, an endogenous non-toxic molecule and an energetic substrate of the neoglucogenesis process, could be, under specific circumstances, the source of lactic acidosis and induce such deleterious consequences for organ function [21].

This discrepancy could be explained by the Stewart-Fencl physicochemical approach. In this model, any acid–base modification is a reflection of water dissociation into protons rather than the accumulation of acid *per se*. Thus, all strong acids such as lactic acid are completely dissociated at physiological pH in water, thus generating protons [22]. The strong ion difference (SID) is the difference between the sums of the concentrations of the strong cations and strong anions:

$$ \left[\mathrm{S}\mathrm{I}\mathrm{D}\right] = \left[{\mathrm{Na}}^{+}\right] + \left[{\mathrm{K}}^{+}\right] + \left[{\mathrm{Ca}}^{2+}\right] + \left[{\mathrm{Mg}}^{2+}\right] - \left[{\mathrm{Cl}}^{-}\right] - \left[\mathrm{Other}\ \mathrm{strong}\ \mathrm{anions}\right] $$

In the shock state, therefore, the increase in lactate production and the decrease in the efficiency of lactate clearance at the cellular level result in a net increase in lactate and a drop in intracellular pH. To maintain intracellular pH in physiological ranges (7.15 to 7.25), mono-carboxylate transporters extrude lactate and H^+^ through the plasma membrane [23]. Following the Stewart model, the accumulated extracellular lactate reduces SID and lowers extracellular pH by proton generation.

Thus, according to the Stewart approach, at constant value of chloremia, albuminemia and PCO_2_, accumulation of lactate is always associated with lactic acidosis [24]. However, lactic acidosis with coexisting metabolic acid–base disturbances (with dyschloremia, adapted or non-adapted PCO_2_ , and so on) is by far the most common situation [25].

## Is lactic acidosis harmful for cardiovascular function?

Regulation of the intracellular and extracellular pH of cardiac or vascular smooth muscle cells (VSMCs) is essential for the maintenance of a stable hemodynamic status. As alluded to above, regardless of the mechanism involved, lactate generation in shock states leads to a drop in intracellular and extracellular pH and most often to hemodynamic failure. Whether this severe lactic acidosis is a causal contributor to multiple organ failure or simply a biomarker of the patient’s critical state remains an ongoing debate. In this situation, severe lactic acidosis in experimental studies always causes negative effects on cardiovascular function while its correction negates its protective effects [26]. Again, no human study has so far clearly replicated these same experimental findings.

In the following sections, the hemodynamic consequences of lactic acidosis at both the cellular and functional level are described. However, a cautious interpretation must be made of the available data. In fact, a large portion of the published experimental data used non-organic acid to induce metabolic acidosis. Therefore, the number of relevant and published studies centered on the effects of acidosis induced by an accumulation of extracellular lactate reducing SID and lowering extracellular pH by proton generation is somewhat limited. By hypothetical reasoning, it is usually accepted that the effects of lactic acidosis may overlap with those of metabolic acidosis. Nevertheless, in the following sections, the manner in which acidosis is induced will be specified for each included reference; that is, the hypoxic lactic acidosis model (LAM) or non-organic acidosis model (NOAM). It is likely that some of the hemodynamic effects reported in hypoxic LAMs are also induced in part by hypoxia [27]. However, the latter remains the most widely used model to induce an endogenous and homogenous shock-associated lactic acidosis. In addition, when acidosis is induced via NOAM, the cited text will systematically carry the mention that the study involved metabolic acidosis including lactic acidosis.

### Lactic acidosis and myocardial cell dysfunction

**Figure 1:**
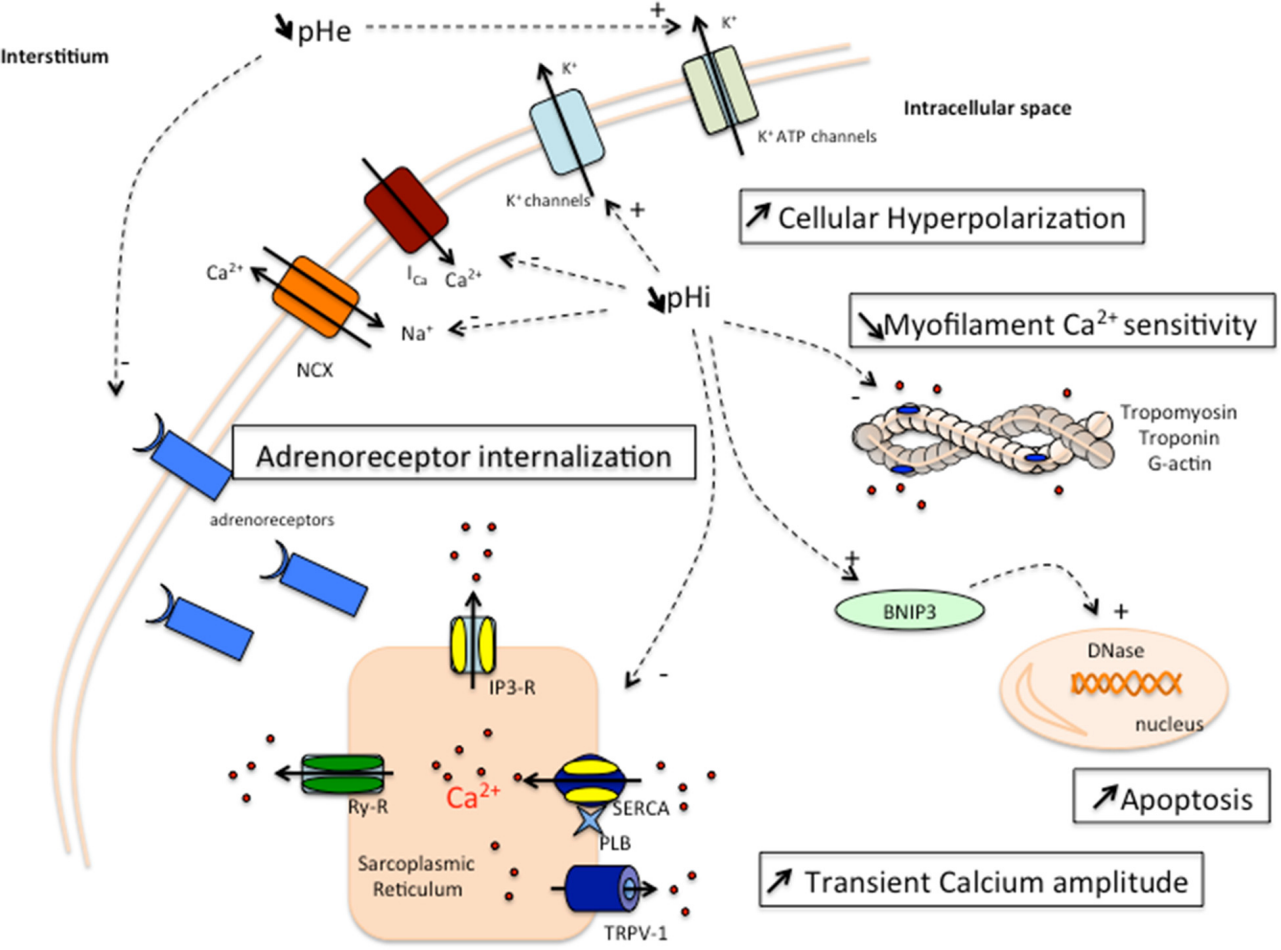
**Description of the principal pathophysiological effects of severe metabolic acidosis with pH <7.2 on a muscle cell.** Transient calcium amplitude: the increase in Ca^2+^ transient amplitude is the net consequence of the inhibitory effect of low intracellular pH on RyRs, NCX and I_Ca_, and the stimulatory effects of low intracellular pH on NHE, NBC, TRVP-1 and sarcoplasmic reticulum Ca^2^. Myofilament Ca^2+^ sensitivity: due to the low intracellular pH, Ca^2+^ binding to troponin is altered and myofilament Ca^2+^ sensitivity decreased. Cellular hyperpolarization: intracellular acidosis also enhances hyperpolarization through K^+^ extrusion. Apoptosis: intracellular acidosis has stimulatory effects on BNIP3, promoting apoptosis. Adrenoreceptors: extracellular and intracellular acidosis reduces the number of adrenoreceptors on the cell membrane. Ica, L-type Ca^2+^ channel; IP3-R, inositol-1,4,5-triphosphate receptor; NBC, Na^+^/HCO_3_^−^ co-transport; NCX, Na^+^/Ca^2+^ exchange; NHE, Na^+^/H^+^ exchange; pHe, extracellular pH; pHi, intracellular pH; PLB, phospholamban; Ry-R, ryanodine receptor; SERCA, sarco/endoplasmic reticulum Ca^2+^-ATPase; SR, sarcoplasmic reticulum; TRVP-1, transient receptor potential channels-1.

In cardiac cells, the drop in intracellular pH has a considerable impact on the amplitude of the systolic calcium transient and the subsequent excitation-contraction coupling pathway (NOAM) [28] (Figure 1). The net impact of intracellular lactic acidosis is an increase in the calcium transient amplitude due to increased sarcoplasmic reticulum Ca^2+^ content despite a decrease in fractional release (NOAM) [29]. Three major mechanisms globally regulate the sarcoplasmic reticulum Ca^2+^ concentration: 1) desensitization of the ryanodine receptor and decreased calcium release by the sarcoplasmic reticulum (LAM) [29,30]; 2) extrusion of H^+^ via Na^+^/H^+^ exchange, increasing the intracellular Na^+^ concentration, which stimulates Na^+^/Ca^2+^ exchange and further increases the intra-cytoplasmic Ca^2+^ concentration (NOAM and LAM) [31,32]; and 3) inhibition of sarco/endoplasmic reticulum Ca^2+^-ATPase (SERCA) but also phosphorylation of phospholamban, which in turn increases Ca^2+^ uptake from the cytosol by SERCA (NOAM and LAM) [33,34].

Paradoxically, activation of the Na^+^/H^+^ exchanger (NHE) in order to increase intracellular pH also has the potential of giving rise to deleterious increases in cytosolic calcium and sodium concentrations [35].

Intra- and extracellular lactic acidosis also have an impact on all action potential mechanisms; that is, the delicate balance between inward and outward currents. The current literature, most of which is experimental, reports various effects on action potential depending on the degree and method of acidosis used. One of the most studied aspects in acidosis is the consequence of a change in calcium transient on the action potential and its clinical relevance to cardiac arrhythmias. Schematically, intracellular metabolic acidosis, including of lactic acid origin, as seen above, increases the intracellular calcium transient but also its alternans, which impacts repolarization alternans susceptibility (NOAM) [36].

It has long been known that a drop in intracellular pH not only changes the calcium transient amplitude but also alters Ca^2+^ binding to troponin C (NOAM and LAM) [37,38]. This effect occurs not only in severe but also mild acute metabolic acidosis (LAM) [39].

The consequences of lactic acidosis on the apoptosis pathway have been widely investigated in myocardial ischemia models but poorly studied in sepsis-induced cardiovascular dysfunction. Among the numerous studied mechanisms, BNIP3, a member of the Bcl-2 pro-apoptotic protein family, mainly contributes to cardiomyocyte cell death (LAM) [40,41]. Undetectable in healthy cells, hypoxic conditions such as myocardial infarction or trauma-hemorrhage injury promote *BNIP3* gene expression and its accumulation in the cytoplasm. However, only the association of hypoxia with intracellular lactic acidosis induces the activation of the death pathway (LAM) [42]. Under intracellular lactic acidosis conditions, BNIP3 translocates into the mitochondrial membrane, thereby opening the mitochondrial permeability transition pore. Thereafter, mitochondria subsequently release pro-apoptotic factors (cytochrome c, apoptosis-inducing factors, and so on) that stimulate nuclear translocation of DNase without activation of caspases (LAM) [40]. Other mechanisms, described in experimental endothelial cell models of ischemic acidosis, involve accumulation of cytosolic Ca^2+^ leading to the activation of caspases and apoptosis (LAM) [43]. Finally, the drop in extracellular pH also reduces the number of beta-adrenoreceptors on myocardial cell surfaces (NOAM) [44].

### Lactic acidosis and vascular smooth muscle cell dysfunction

Metabolic acidosis, including lactic acidosis, induces significant effects on VSMCs in close relationship with endothelial cells (Figure 1). Lactic acidosis initiates multiple cascades of intracellular signaling reactions in both endothelial cells and VSMCs.

As in myocardial cells, intracellular metabolic acidosis, including lactic acidosis, also alters the calcium transient and reduces the number of adrenoreceptors on the cell surface (NOAM) [11,45]. More specifically, lactic acidosis induces vascular smooth muscle relaxation via the opening of ATP-sensitive potassium channels (NOAM and LAM) [46,47].

Widely demonstrated, metabolic acidosis, including lactic acidosis, also leads to the expression of inducible nitric oxide synthase in endothelium and VSMCs. Overproduction of nitric oxide has a direct vasodilator effect on VSMCs (NOAM and LAM) [48-51].

Intracellular pH regulation in VSMCs is partly dependent on transmembrane movement of acid/base equivalents. Three well-characterized channels are known to be involved in intracellular pH regulation: 1) the NHE, which extrudes proton in exchange for sodium (NOAM); 2) the Cl^−^/HCO_3_^−^ exchanger, which maintains a high concentration of intracellular chloride and is activated in response to intracellular alkalinization (NOAM); and 3) Na^+^/HCO_3_^−^ co-transport, which is also stimulated by a drop in intracellular pH (NOAM) [52-55].

### Functional myocardial consequences of severe lactic acidosis

**Figure 2:**
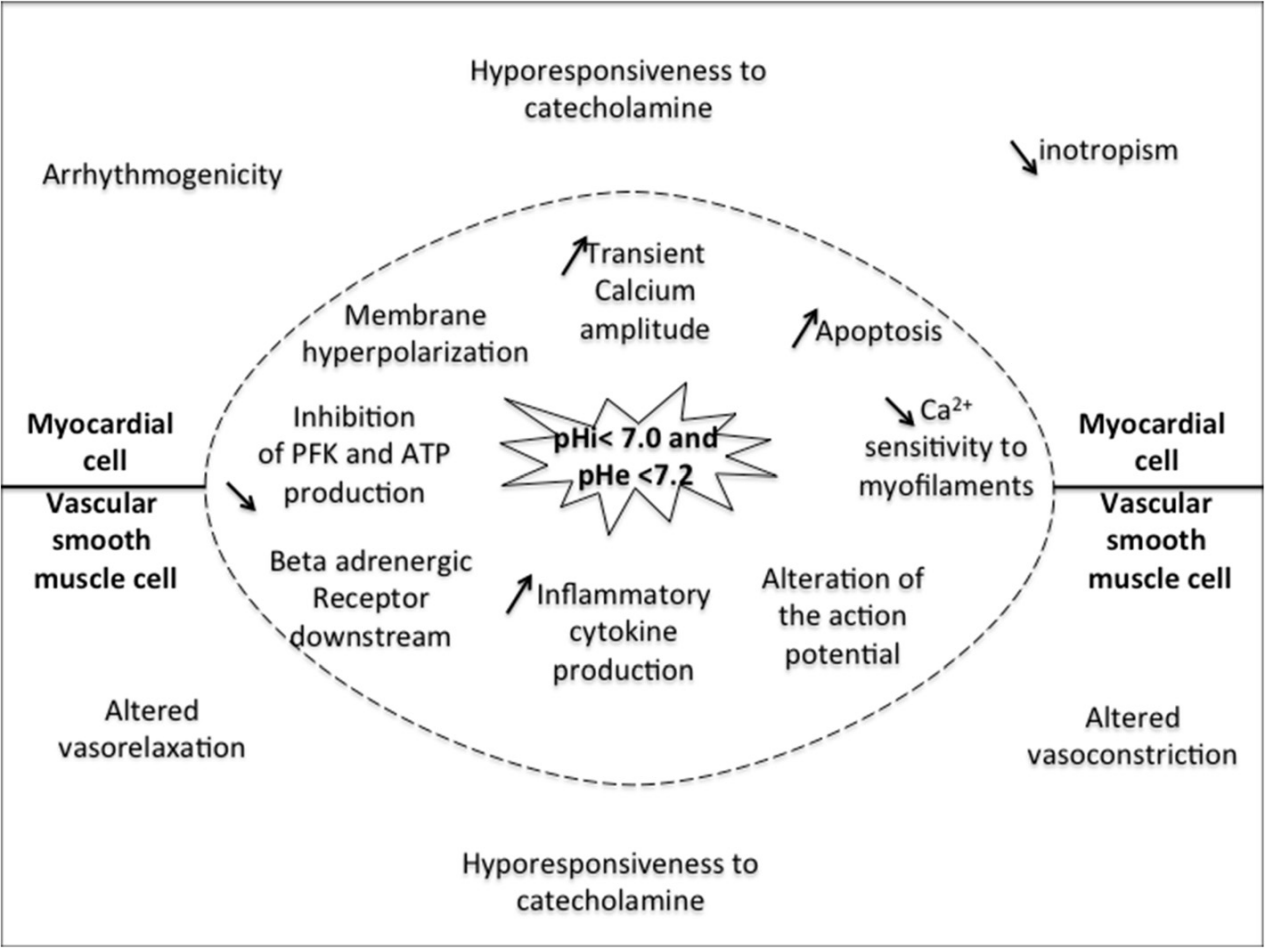
**Schematic representation of cellular and functional consequences in myocardial and vascular smooth muscle cells in instances of severe lactic acidosis.** The same mechanisms are involved in both cell types but with specific functional consequences. PFK, phospho-fructo-kinase; pHe, extracellular pH; pHi, intracellular pH.

Lactic acidosis has been known for over 50 years to impair cardiac function [56-59] (Figure 2). In isolated rabbit hearts, Berger and colleagues elegantly demonstrated that lactic acidosis depressed ventricular elastance (LAM) [9]. In an *in vivo* model of severe lactic acidosis induced by hemorrhagic shock, inotropism assessed by a conductance catheter was also altered (LAM) [60]. However, human studies are lacking on this specific subject. A recent study on isolated human ventricular trabeculae showed that a mild metabolic acidosis, including lactic acidosis, reduced both contractility and beta-adrenergic response to isoproterenol (NOAM) [39]. Other experimental studies also confirm the metabolic/lactic acidosis-induced hyporesponsiveness to inotropic agents (NOAM) [44,61]. Despite sparse data, metabolic acidosis, including lactic acidosis, also appears to depress myocardial relaxation assessed in isolated heart or by echocardiography (NOAM) [62,63].

Conversely, the literature is extensive on targeting of the pathophysiology of metabolic acidosis in cardiac arrhythmias, mainly in ischemia-reperfusion models. By increasing cellular calcium transient alternans, metabolic acidosis, including lactic acidosis, also promotes repolarization alternans susceptibility (NOAM) [36]. Indeed, repolarization wave alternans has been shown to be a prognostic marker for the occurrence of severe arrhythmias such as ventricular fibrillation [64,65].

### Functional vascular consequences of severe lactic acidosis

Both *in vivo* and *ex vivo* experimental studies have clearly demonstrated that severe lactic acidosis is associated with major deleterious vascular consequences, although these effects have not been formally demonstrated in humans (Figure 2). Experimentally, the reduction in contractile response to increasing doses of phenylephrine defines vascular hyporesponsiveness to vasopressors. For example, in a myography chamber, segments of healthy rat arterial vessels exposed to a severe acidotic medium displayed a reduced contractile response to phenylephrine or potassium (NOAM and LAM) [10,60,66,67]. However, vascular response assessed by changes in vascular tone to catecholamines does not necessary translate into a resulting change in mean arterial pressure. Indeed, arterial pressure is measured in compliance vessels, which represent only 30% of systemic vascular resistances. Relaxation of arterial vessels is also decreased by severe metabolic acidosis, including lactic acidosis, although this aspect is less well documented (NOAM) [68].

There is no clear, published definition of vascular hyporesponsiveness to vasopressor therapy in clinical practice. The inability to increase arterial pressure despite high vasopressor doses in shock patients could be one suggested definition. However, there are currently no available human data using this definition and demonstrating a direct imputable link between lactic acidosis and impaired vascular function.

### Potential beneficial effect of acidosis related to hyperlactatemia

In addition to the known deleterious effects of acidosis, experimental studies prior to 1980 reported several examples of the beneficial effect of moderate metabolic (including lactic) acidosis on hemodynamics [69]. Thus, recent literature has emerged regarding the potential favorable effects of mild acidosis, particularly in the setting of cardiac surgery in order to reduce the harmful effects of postoperative ischemia-reperfusion.

For instance, in a canine model of coronary ischemic-reperfusion syndrome, Fujita and colleagues [70] reported that prolonged transient acidosis during early reperfusion was found to reduce myocardial injuries (LAM). These unintuitive effects of acidosis may be related to the decrease in calcium overload, which attenuates myocardial consumption [71]. Acidosis also attenuates neutrophil activation and free radical generation [72]. Moreover, nitric oxide and adenosine release are enhanced, contributing to protecting the heart from reperfusion injury (LAM) [73,74].

Under acidotic conditions, the sigmoid HbO_2_ dissociation curve undergoes a rightward shift, resulting in a decrease in SaO_2_ and an increase in tissue O_2_ delivery. Due to the shape of the HbO_2_ curve, the effects of such a shift are usually insignificant at normal PO_2_ levels but are critical at low PO_2_ levels [75]. *In vitro* studies have determined that acidosis activates ATP-sensitive potassium channels leading to vasorelaxion via membrane hyperpolarization [76]. This in turn may increase microvascular flow and thus contribute to the reperfusion syndrome. Finally, even if counterintuitive, acidosis reduces ATP production and energy expenditure, which could lead, at a cellular level, to a protective effect against death [77].

## Is there any proven benefit of systemic alkalinization in severe metabolic (including lactic) acidosis?

Literature regarding the potential beneficial effect of alkalinization in correcting metabolic acidosis is controversial. As reported above, severe lactic acidosis with pH ≤7.15 appears to be experimentally detrimental for organ functions. Consequently, even if not formally demonstrated in clinical trials, it would appear reasonable to quickly correct the pH in order to restore cellular functions. In the absence of conclusive clinical studies, however, most of the following treatment options are consequently based on experimental data.

### Sodium bicarbonate

Sodium bicarbonate has been removed from the treatment algorithm in advanced cardiac life support [78]. The Surviving Sepsis Campaign also recommends against the use of sodium bicarbonate therapy for the purpose of improving hemodynamics or reducing vasopressor requirements in patients with severe lactic acidosis with pH >7.15 [2]. Despite these strong guidelines, in the most recent survey on this topic, 67% of intensivists recommend administration of base to patients with metabolic acidosis, including lactic acidosis. The blood pH at which base therapy should be initiated remains nonetheless controversial. Thirty-seven percent of these intensivists continue to begin symptomatic treatment of metabolic acidosis for a pH ≥7.1 [4]. Such discrepancy between the literature and bedside practice warrants further explanation.

Clinical studies investigating sodium bicarbonate therapy in situations of severe lactic acidosis have always reported an increase in extracellular pH whereas experimental data are more divergent. By contrast, intracellular pH always decreases after sodium bicarbonate administration (Table 1). The main explanation for this so-called paradoxical intracellular acidosis is based on the reaction of sodium bicarbonate with a proton to form water and carbon dioxide:

$$ {{\mathrm{H}\mathrm{CO}}_3}^{-} + {\mathrm{H}}^{+}\iff {\mathrm{H}}_2\mathrm{O} + {\mathrm{CO}}_2 $$

**Table 1:** **Reported effects of Sodium Bicarbonate on intracellular and extracellular pH, hemodynamics and mortality in** ***in vivo*** **experimental and clinical studies**

**Study**	**Experimental (E) or human (H)**	**Methodology (intervention/subjects/protocol/measurements)**	**Increased PaCO**_2_ **after alkalinization in HCO**_3_^**−**^ **group?**	**Decreased or unchanged pHe or pHi after alkalinization in HCO**_3_^**−**^ **group or compared with other groups?**	**Shock associated lactic acidosis?**	**Positive effects of sodium bicarbonate on hemodynamics (arterial pressure/ cardiac index)** ^**a**^	**Positive impact of sodium bicarbonate on mortality** ^**b**^
**Kim** ***et al*** **. 2013** [112]	H	Retrospective. 103 patients with lactic acidosis. Effects of HCO_3_^−^ on survival	NA	NA	Yes	NA	NA
**Wilson** ***et al*** **. 2013** [81]	H	Retrospective series. Severe acidotic trauma patients. Effects of HCO_3_^−^ on survival, PaCO_2_, pH	Yes	pHe: no	Yes	NA	NA
				pHi: NA.			
**Levraut** ***et al*** **. 2000** [113]	H	Mild metabolic acidosis in non-shock patients. Effects of a bicarbonate load on CO_2_ generation depending on non-bicarbonate buffer	Yes	pHe: no	No	NA	NA
				pHi: no			
**Nielsen** ***et al*** **. 2002** [114]	H	5-minute rhythmic handgrip to provoke intracellular acidosis. Healthy subjects. HCO_3_^−^ vs. saline. Effect on arterial pH, and muscle pHi, PaCO_2_	Yes	pHe: no	No	NA	NA
				pHi: no			
**Nakashima** ***et al*** **. 1996** [115]	H	Healthy subjects. Effects of HCO_3_^−^ infusion on cerebral blood flow, PaCO_2_ and pHi	Yes	pHe: no	No	NA	NA
				pHi: yes			
**Leung** ***et al*** **. 1994** [100]	H	Metabolic acidosis in patients undergoing surgery. HCO_3_^−^ vs. carbicarb. Effects on pHe, hemodynamics	NA	pHe: no	No	No	NA
				pHi: NA			
**Mark** ***et al*** **. 1993** [116]	H	Intraoperative mild acidosis. HCO_3_^−^ vs. saline. Effects on PaCO_2_, pH, hemodynamics	Yes	pHe: no	No	NA	NA
				pHi: NA			
**Fanconi** ***et al*** **. 1993** [117]	H	Neonatal acidosis. HCO_3_^−^ before-after study. Effect on hemodynamics, pH, PaCO_2_, PtCO_2_	Yes	pHe: no	Yes	Yes	NA
				pHi: NA			
**Mathieu** ***et al*** **. 1991** [92]	H	Septic shock. HCO_3_^−^ vs. saline. Effect on arterial pH, PaCO_2_, hemodynamics	Yes	pHe: no	Yes	No	NA
				pHi: NA			
**Cooper** ***et al*** **. 1990** [89]	H	Septic shock. HCO_3_^−^ vs. saline. Effect on arterial pH, PaCO_2_, hemodynamics	Yes	pHe: no	Yes	No	NA
				pHi: NA			
**Bersin** ***et al*** **. 1989** [118]	H	Congestive heart disease. HCO_3_^−^ vs. saline. Effect on acidosis, PaCO_2_, hemodynamics (myocardial oxygen consumption)	Yes	pHe: no	No	No	NA
				pHi: NA			
**Kimmoun** ***et al*** **. 2014** [60]	E	Hemorrhagic shock. Rats. HCO_3_^−^ with calcium adjunction and increased respiratory rate. Effect on pHe, muscle pHi, hemodynamics	No	pHe: No	Yes	Yes	NA
				pHi: No			
**Valenza** ***et al*** **. 2012** [84]	E	Lactic acid infusion. Rats. Lactic acidosis vs. lactic acidosis + sodium bicarbonate. Effects on hemodynamics, pHe, lactate, phosphofructokinase.	Yes	pHe: No	No	No	NA
				pHi: NA			
**Beech** ***et al*** **. 1994** [87]	E	Hypovolemic shock. Rats. Carbicarb vs. HCO_3_^−^. Muscle pHi, PaCO2 and hemodynamics	Yes	pHe: No	Yes	No	NA
				pHi: Yes			
**Bollaert** ***et al*** **. 1994** [79]	E	Endotoxinic shock. Rats. HCO_3_^−^ vs. saline. Effect on arterial pH, PaCO_2,_ muscle pHi, hemodynamics	Yes	pHe: No	Yes	No	NA
				pHi: Yes			
**Rhee** ***et al*** **. 1993** [83]	E	Hypoxic lactic acidosis. Mongrel dogs. HCO_3_^−^ vs. Carbicarb vs. saline. Effects on PaCO_2_, hemodynamics	Yes	pHe: Yes	Yes	No	NA
				pHi: Yes			
**Cooper** ***et al*** **. 1993** [88]	E	L-lactic infusion. Pigs. HCO_3_^−^ vs. saline. Effects on pH, hemodynamics	Per protocol ventilation adjustment	pHe: No	Yes	No	NA
				pHi: NA.			
**Shapiro** ***et al*** **. 1990** [119]	E	Ammonium chloride-induced metabolic acidosis. HCO_3_^−^ vs. Carbicarb. Effects on PaCO_2_, pHe, hepatic pHi, hemodynamics	Yes	pHe: No	No	No	NA
				pHi: Yes			
**Dimlich** ***et al*** **. 1988** [120]	E	Low-flow-induced lactic acidosis. Rats. HCO_3_^−^ vs. NaDCA vs. NaCl. Effects on pH, lactatemia	NA	pHe: No	Yes	No	NA
				pHi: NA.			
**Iberti** ***et al*** **. 1988** [91]	E	Hemorrhagic shock. Dogs. HCO_3_^−^ vs. saline. Effect on hemodynamics, pH, PaCO_2_	Yes	pHe: Yes	Yes	No	NA
				pHi: NA.			
**Hope** ***et al*** **. 1988** [121]	E	Incomplete cerebral ischemia in lamb. Effects of glucose and HCO_3_^−^ on cerebral pHi, PaCO_2_ and PtiCO_2_	Yes	pHe: No	No	NA	NA
				pHi: Yes			
**Sessler** ***et al*** **. 1987** [122]	E	Lactic acidosis treatment in neonatal rabbits. Effect of HCO_3_^−^ on pHi and pHe and PaCO_2_	Yes	pHe: no	Yes	NA	NA
				pHi: no			
**Graf** ***et al*** **. 1985** [90]	E	Hypoxic lactic acidosis. Dogs. HCO_3_^−^ vs. saline vs. no therapy. Effects on pHe and hemodynamics	NA	pHe: yes	Yes	No	No
				pHi: NA			
**Graf** ***et al*** **. 1985** [123]	E	Hypoxic lactic acidosis. Dogs. HCO_3_^−^ vs. saline. Effects on pHe and hepatic pHi	Yes	pHe: yes	Yes	No	No
				pHi: yes			
**Arieff** ***et al*** **. 1982** [82]	E	Phenformin-induced lactic acidosis. Dogs. HCO_3_^−^ vs. saline vs. placebo. Effects on pHe, pHi, hemodynamics	NA	pHe: yes	Yes	No	No
				pHi: yes			

^a^Only applicable in comparative studies with critical patients or experimental models. ^b^Only applicable in comparative studies with critical patients or experimental models. NA, not applicable; pHe, extracellular pH; pHi, intracellular pH.

This large generation of carbon dioxide has been observed in all previous clinical and experimental studies (Table 1). Carbon dioxide rapidly diffuses across the cell membrane, resulting in intracellular hypercapnic acidosis, which impairs organ function [79-81]. The rise in carbon dioxide partial pressure also increases hemoglobin affinity for oxygen and may, therefore, decrease oxygen delivery. The rise in lactate after bicarbonate administration, noted in many studies, could be the consequence of this impaired oxygen delivery to tissues [82,83]. Furthermore, upon administration of bicarbonate, the generated alkalosis favors glucose metabolism. Consequently, glucose levels decrease more rapidly than lactate levels, thus worsening hyperlactatemia. Moreover, compared with low pH levels, lactate oxidation is reduced when pH increases [84]. Moreover, bicarbonate decreases ionized calcium, which, as discussed above, plays a pivotal role in cellular contraction [60,85,86]. It is thus not surprising that experimental and human studies assessing the effects of sodium bicarbonate effects in shock patients with severe lactic acidosis have not shown any improvement in cardiovascular function [82,83,87-92]. Therefore, as suggested by Boyd and Walley [85], it is likely that all potential beneficial effects of sodium bicarbonate therapy have been dampened by these two major side effects. A recent experimental study determined the cardiovascular effects of an adapted sodium bicarbonate therapy that included the prevention of both carbon dioxide increase and ionized calcium decrease in a model of severe lactic acidosis induced by hemorrhagic shock. The main finding was that bicarbonate therapy in this specific setting improved both cardiac and vascular function in addition to raising intra- and extracellular pH [60].

In light of these data, bicarbonate therapy might be useful in critical situations in the expectation of specific etiological treatment efficacy. Translating these results to the clinical bedside needs to be confirmed in clinical trials aimed at determining which patients may benefit from this strategy. The design of such a study would likely be difficult to develop and entail several difficulties.

### THAM and carbicarb

Given the side effects of sodium bicarbonate, other alkali therapies have been developed. THAM (tris-hydroxymethyl-aminomethane) and carbicarb (equimolar mixture of sodium bicarbonate and sodium carbonate) constitute the two most prominent molecules in this context.

THAM buffers protons and particularly carbon dioxide, as described in the following reactions:

$$ \begin{array}{l}\mathrm{R}-{\mathrm{NH}}_2 + {\mathrm{H}}_2\mathrm{O} + {\mathrm{CO}}_2\iff \mathrm{R}-{{\mathrm{NH}}_3}^{+} + {{\mathrm{H}\mathrm{CO}}_3}^{-}\\ {}\mathrm{R}-{\mathrm{NH}}_2 + {\mathrm{H}}^{+} + {\mathrm{lactate}}^{-}\iff {{\mathrm{RNH}}_3}^{+} + {\mathrm{lactate}}^{-}\end{array} $$

THAM diffuses into the intracellular space in non-ionized form and is able to raise intracellular pH [93]. In theory, its use should represent an interesting option; however, its effects on pH are limited over time by its immediate urinary excretion. Due to its toxicity (hyperkalemia), its usefulness in the critical care setting is reduced in instances of significant renal impairment with a glomerular filtration rate under 30 ml.minute^−1^.

In experimental studies, the buffering capacity of THAM is comparable to that of bicarbonate but without the generation of carbon dioxide [94]. In a blood-perfused isolated heart model with a pH lowered to 7.0, THAM also partially corrects pH and improves myocardial contractility and relaxation. Interestingly, a mixture of sodium bicarbonate with THAM has been shown to enable a complete recovery of pH, improve myocardial function and prevent intracellular paradoxical acidosis [95]. Clinical trials in critical patients with relevant lactic acidosis assessing the efficacy and/or the hemodynamic effects of THAM versus other alkali therapies are alas methodologically poor. The most recent randomized study included only 18 patients with mild metabolic (including lactic) acidosis. The authors concluded that THAM and sodium bicarbonate had similar alkalinizing effects [96]. More robust randomized and controlled studies assessing cardiovascular function would be of valuable interest in determining which patients may benefit from this therapeutic option.

THAM also has considerable side effects, including hepatic failure, hyperkalemia, hypoglycemia and, if the molecule is infused via a peripheral venous access, a potential risk of extravasation and cutaneous necrosis [97]. Hence, although an interesting agent, its usefulness remains questionable, particularly in the case of acute renal failure, which is a frequent clinical setting in the intensive care unit.

Carbicarb was also developed in order to reduce carbon dioxide generation. This molecule, in theory, would limit the drop in intracellular pH compared with that induced by a bicarbonate load. Experimental studies in dogs comparing carbicarb versus bicarbonate therapy showed the superiority of carbicarb in improving intracellular pH and cardiac output [98,99]. In patients who developed metabolic acidosis while undergoing major surgery, carbicarb demonstrated its superiority compared with sodium bicarbonate therapy in improving cardiac output with no deleterious side effects [100]. As above, however, no relevant clinical trials have been performed in situations of more severe acidosis.

### Renal replacement therapy

While sodium bicarbonate remains a controversial therapy in instances of severe lactic acidosis, it is somewhat remarkable that renal replacement therapy (RRT), which also provides a significant amount of bicarbonate buffer, is very rarely discussed.

Similarly to the modified bicarbonate therapy, RRT could be initiated in case of an uncontrolled shock state mainly attributed to concomitant severe lactic acidosis. Two modalities could be envisaged: intermittent hemodialysis (IHD) and continuous veno-venous hemofiltration (CVVHF). Compared with IHD, CVVHF corrects pH more rapidly. Moreover, because of its permanent rebalancing effects on acid–base status, CVVHF therapy is preferred to IHD [101]. Thus, CVVHF should be preferred to IHD.

Many studies have also compared various buffer solutions. Under physiological conditions, acetate and lactate are metabolized into bicarbonate and carbon dioxide. However, during shock states, the metabolic rate of lactate or acetate may be reduced due to liver failure. As demonstrated by Tan and colleagues [102], even without hepatic failure, RRT with a lactate buffer solution induces iatrogenic hyperlactatemia associated with an acidifying effect. Accordingly, during the shock state or in instances of multiple organ failure, including hepatic dysfunction, the use of a bicarbonate buffer solution is warranted [103].

The optimal intensity of CVVHF therapy is unclear, particularly for the correction of the acid–base status. In critically ill patients with acute kidney injury, however, high-volume CVVHF does not reduce mortality at 90 days [104]. Finally, a recent study demonstrated that, in patients with mild metabolic, mainly non-lactic acidosis and acute renal failure, standard and high-volume CVVHF had similar effects on acid base status [105]. As suggested by the Surviving Sepsis Campaign guidelines, a typical dose of 20 to 25 ml.kg^−1^.h^−1^ is recommended [2].

Although efficient in normalizing pH, beneficial effects of CVVHF on hemodynamics are not yet convincing. Indeed, trials on the beneficial hemodynamic effects of CVVHF are mostly non-randomized with low statistical power [105-108]. Furthermore, the effects of RRT on intracellular pH are poorly described in the literature.

## Therapeutic perspectives

As presented above, alkalinization with base does not necessarily result in improved cellular or hemodynamic functions and survival rate [25]. Targeting the pH regulatory protein NHE could represent an innovative approach to lactic acidosis management. NHE activation results in intracellular sodium and calcium overloads, which exert deleterious effects on cardiovascular function [109]. A recent experimental study, with a relevant LAM, demonstrated that sabiporide improved myocardial function, reduced systemic inflammation and prevented multiple organ failure [110]. An ensuing experimental study with a clinically relevant model of septic shock also demonstrated similar effects [111].

## Conclusion

Deleterious hemodynamic effects of severe lactic acidosis are largely suggested by experimental data, although not fully confirmed by human studies. Pending the effectiveness of an etiological treatment, there is no efficient and validated symptomatic therapy at hand to correct a life-threatening metabolic acidosis. Upcoming research in this field should be focused on the optimal strategy to treat severe metabolic acidosis, including symptomatic therapy.

## Abbreviations

CVVHF: Continuous veno-venous hemofiltration
IHD: Intermittent hemodialysis
LAM: Lactic acidosis model
NHE: Na^+^/H^+^ exchanger
NOAM: Non-organic acidosis model
RRT: Renal replacement therapy
SERCA: Sarco/endoplasmic reticulum Ca^2+^-ATPase
SID: Strong ion difference
THAM: Tris-hydroxymethyl-aminomethane
VSMC: Vascular smooth muscle cell

## References

[1] Cecconi M, De Backer D, Antonelli M, Beale R, Bakker J, Hofer C (2014). Consensus on circulatory shock and hemodynamic monitoring. Task force of the European Society of Intensive Care Medicine. Intensive Care Med.

[2] Dellinger RP, Levy MM, Rhodes A, Annane D, Gerlach H, Opal SM (2013). Surviving sepsis campaign: international guidelines for management of severe sepsis and septic shock, 2012. Crit Care Med.

[3] Morris CG, Low J (2008). Metabolic acidosis in the critically ill: part 1. Classification and pathophysiology. Anaesthesia.

[4] Kraut JA, Kurtz I (2006). Use of base in the treatment of acute severe organic acidosis by nephrologists and critical care physicians: results of an online survey. Clin Exp Nephrol.

[5] Noritomi DT, Soriano FG, Kellum JA, Cappi SB, Biselli PJ, Liborio AB, Park M (2009). Metabolic acidosis in patients with severe sepsis and septic shock: a longitudinal quantitative study. Crit Care Med.

[6] Smith I, Kumar P, Molloy S, Rhodes A, Newman PJ, Grounds RM, Bennett ED (2001). Base excess and lactate as prognostic indicators for patients admitted to intensive care. Intensive Care Med.

[7] Bakker J, Nijsten MW, Jansen TC (2013). Clinical use of lactate monitoring in critically ill patients. Ann Intensive Care.

[8] Friesecke S, Abel P, Roser M, Felix SB, Runge S (2010). Outcome of severe lactic acidosis associated with metformin accumulation. Crit Care.

[9] Berger DS, Fellner SK, Robinson KA, Vlasica K, Godoy IE, Shroff SG (1999). Disparate effects of three types of extracellular acidosis on left ventricular function. Am J Physiol.

[10] Otter D, Austin C (2000). Simultaneous monitoring of vascular contractility, intracellular pH and intracellular calcium in isolated rat mesenteric arteries; effects of weak bases. Exp Physiol.

[11] Levy B, Collin S, Sennoun N, Ducrocq N, Kimmoun A, Asfar P (2010). Vascular hyporesponsiveness to vasopressors in septic shock: from bench to bedside. Intensive Care Med.

[12] Jung B, Rimmele T, Le Goff C, Chanques G, Corne P, Jonquet O (2011). Severe metabolic or mixed acidemia on intensive care unit admission: incidence, prognosis and administration of buffer therapy. A prospective, multiple-center study. Crit Care.

[13] Kajbaf F, Lalau JD (2014). Mortality rate in so-called “metformin-associated lactic acidosis”: a review of the data since the 1960s. Pharmacoepidemiol Drug Saf.

[14] Barbee RW, Reynolds PS, Ward KR (2010). Assessing shock resuscitation strategies by oxygen debt repayment. Shock.

[15] Richter EA, Kiens B, Saltin B, Christensen NJ, Savard G (1988). Skeletal muscle glucose uptake during dynamic exercise in humans: role of muscle mass. Am J Physiol.

[16] Levy B, Gibot S, Franck P, Cravoisy A, Bollaert PE (2005). Relation between muscle Na + K+ ATPase activity and raised lactate concentrations in septic shock: a prospective study. Lancet.

[17] Levy B (2006). Lactate and shock state: the metabolic view. Curr Opin Crit Care.

[18] Wutrich Y, Barraud D, Conrad M, Cravoisy-Popovic A, Nace L, Bollaert PE (2010). Early increase in arterial lactate concentration under epinephrine infusion is associated with a better prognosis during shock. Shock.

[19] Juneja D, Singh O, Dang R (2011). Admission hyperlactatemia: causes, incidence, and impact on outcome of patients admitted in a general medical intensive care unit. J Crit Care.

[20] Tsai MH, Chen YC, Lien JM, Tian YC, Peng YS, Fang JT (2008). Hemodynamics and metabolic studies on septic shock in patients with acute liver failure. J Crit Care.

[21] Garcia-Alvarez M, Marik P, Bellomo R (2014). Stress hyperlactataemia: present understanding and controversy. Lancet Diabetes Endocrinol.

[22] Morris CG, Low J (2008). Metabolic acidosis in the critically ill: part 2. Causes and treatment. Anaesthesia.

[23] Broer S, Schneider HP, Broer A, Rahman B, Hamprecht B, Deitmer JW (1998). Characterization of the monocarboxylate transporter 1 expressed in Xenopus laevis oocytes by changes in cytosolic pH. Biochem J.

[24] Langer T, Carlesso E, Protti A, Monti M, Comini B, Zani L (2012). In vivo conditioning of acid–base equilibrium by crystalloid solutions: an experimental study on pigs. Intensive Care Med.

[25] Kraut JA, Madias NE (2014). Lactic acidosis. N Engl J Med.

[26] Teplinsky K, O’Toole M, Olman M, Walley KR, Wood LD (1990). Effect of lactic acidosis on canine hemodynamics and left ventricular function. Am J Physiol.

[27] Regueira T, Djafarzadeh S, Brandt S, Gorrasi J, Borotto E, Porta F (2012). Oxygen transport and mitochondrial function in porcine septic shock, cardiogenic shock, and hypoxaemia. Acta Anaesthesiol Scand.

[28] Crampin EJ, Smith NP, Langham AE, Clayton RH, Orchard CH (2006). Acidosis in models of cardiac ventricular myocytes. Philos Transact A Math Phys Eng Sci.

[29] Choi HS, Trafford AW, Orchard CH, Eisner DA (2000). The effect of acidosis on systolic Ca2+ and sarcoplasmic reticulum calcium content in isolated rat ventricular myocytes. J Physiol.

[30] Dong LW, Wu LL, Ji Y, Liu MS (2001). Impairment of the ryanodine-sensitive calcium release channels in the cardiac sarcoplasmic reticulum and its underlying mechanism during the hypodynamic phase of sepsis. Shock.

[31] Harrison SM, Frampton JE, McCall E, Boyett MR, Orchard CH (1992). Contraction and intracellular Ca2+, Na+, and H+ during acidosis in rat ventricular myocytes. Am J Physiol.

[32] Sikes PJ, Zhao P, Maass DL, White J, Horton JW (2005). Sodium/hydrogen exchange activity in sepsis and in sepsis complicated by previous injury: 31P and 23Na NMR study. Crit Care Med.

[33] DeSantiago J, Maier LS, Bers DM (2004). Phospholamban is required for CaMKII-dependent recovery of Ca transients and SR Ca reuptake during acidosis in cardiac myocytes. J Mol Cell Cardiol.

[34] Wu LL, Tang C, Dong LW, Liu MS (2002). Altered phospholamban-calcium ATPase interaction in cardiac sarcoplasmic reticulum during the progression of sepsis. Shock.

[35] Wu D, Kraut JA (2011). Potential role of NHE1 (sodium-hydrogen exchanger 1) in the cellular dysfunction of lactic acidosis: implications for treatment. Am J Kidney Dis.

[36] Kapur S, Wasserstrom JA, Kelly JE, Kadish AH, Aistrup GL (2009). Acidosis and ischemia increase cellular Ca2+ transient alternans and repolarization alternans susceptibility in the intact rat heart. Am J Physiol Heart Circ Physiol.

[37] Blanchard EM, Solaro RJ (1984). Inhibition of the activation and troponin calcium binding of dog cardiac myofibrils by acidic pH. Circ Res.

[38] Ming MJ, Hu D, Chen HS, Liu LM, Nan X, Hua CH, Lu RQ (2000). Effect of MCI-154, a calcium sensitizer, on calcium sensitivity of myocardial fibers in endotoxic shock rats. Shock.

[39] Schotola H, Toischer K, Popov AF, Renner A, Schmitto JD, Gummert J (2012). Mild metabolic acidosis impairs the beta-adrenergic response in isolated human failing myocardium. Crit Care.

[40] Graham RM, Frazier DP, Thompson JW, Haliko S, Li H, Wasserlauf BJ (2004). A unique pathway of cardiac myocyte death caused by hypoxia-acidosis. J Exp Biol.

[41] Jian B, Wang D, Chen D, Voss J, Chaudry I, Raju R (2010). Hypoxia-induced alteration of mitochondrial genes in cardiomyocytes: role of Bnip3 and Pdk1. Shock.

[42] Kubasiak LA, Hernandez OM, Bishopric NH, Webster KA (2002). Hypoxia and acidosis activate cardiac myocyte death through the Bcl-2 family protein BNIP3. Proc Natl Acad Sci U S A.

[43] Kumar S, Kasseckert S, Kostin S, Abdallah Y, Schafer C, Kaminski A (2007). Ischemic acidosis causes apoptosis in coronary endothelial cells through activation of caspase-12. Cardiovasc Res.

[44] Marsh JD, Margolis TI, Kim D (1988). Mechanism of diminished contractile response to catecholamines during acidosis. Am J Physiol.

[45] Ives SJ, Andtbacka RH, Noyes RD, Morgan RG, Gifford JR, Park SY (2013). alpha1-Adrenergic responsiveness in human skeletal muscle feed arteries: the impact of reducing extracellular pH. Exp Physiol.

[46] Ishizaka H, Kuo L (1996). Acidosis-induced coronary arteriolar dilation is mediated by ATP-sensitive potassium channels in vascular smooth muscle. Circ Res.

[47] Kuo JH, Chen SJ, Shih CC, Lue WM, Wu CC (2009). Abnormal activation of potassium channels in aortic smooth muscle of rats with peritonitis-induced septic shock. Shock.

[48] Pedoto A, Caruso JE, Nandi J, Oler A, Hoffmann SP, Tassiopoulos AK (1999). Acidosis stimulates nitric oxide production and lung damage in rats. Am J Respir Crit Care Med.

[49] Pedoto A, Nandi J, Oler A, Camporesi EM, Hakim TS, Levine RA (2001). Role of nitric oxide in acidosis-induced intestinal injury in anesthetized rats. J Lab Clin Med.

[50] Fernandes D, Assreuy J (2008). Nitric oxide and vascular reactivity in sepsis. Shock.

[51] Yaghi A, Paterson NA, McCormack DG (1995). Vascular reactivity in sepsis: importance of controls and role of nitric oxide. Am J Respir Crit Care Med.

[52] Kahn AM, Cragoe EJ, Allen JC, Halligan RD, Shelat H (1990). Na(+)-H+ and Na(+)-dependent Cl(−)-HCO3- exchange control pHi in vascular smooth muscle. Am J Physiol.

[53] Little PJ, Neylon CB, Farrelly CA, Weissberg PL, Cragoe EJ, Bobik A (1995). Intracellular pH in vascular smooth muscle: regulation by sodium-hydrogen exchange and multiple sodium dependent HCO3- mechanisms. Cardiovasc Res.

[54] Aalkjaer C, Peng HL (1997). pH and smooth muscle. Acta Physiol Scand.

[55] Boedtkjer E, Praetorius J, Aalkjaer C (2006). NBCn1 (slc4a7) mediates the Na + −dependent bicarbonate transport important for regulation of intracellular pH in mouse vascular smooth muscle cells. Circ Res.

[56] Weil MH, Houle DB, Brown EB, Campbell GS, Heath C (1958). Vasopressor agents; influence of acidosis on cardiac and vascular responsiveness. Calif Med.

[57] Bers DM, Ellis D (1982). Intracellular calcium and sodium activity in sheep heart Purkinje fibres. Effect of changes of external sodium and intracellular pH. Pflugers Arch.

[58] Allen DG, Orchard CH (1983). The effects of changes of pH on intracellular calcium transients in mammalian cardiac muscle. J Physiol.

[59] Orchard CH, Kentish JC (1990). Effects of changes of pH on the contractile function of cardiac muscle. Am J Physiol.

[60] Kimmoun A, Ducrocq N, Sennoun N, Issa K, Strub C, Escanye JM (2014). Efficient extra- and intracellular alkalinization improves cardiovascular functions in severe lactic acidosis induced by hemorrhagic shock. Anesthesiology.

[61] Hagiya K, Takahashi H, Isaka Y, Inomata S, Tanaka M (2013). Influence of acidosis on cardiotonic effects of colforsin and epinephrine: a dose–response study. J Cardiothorac Vasc Anesthesia.

[62] McCaul CL, McNamara P, Engelberts D, Slorach C, Hornberger LK, Kavanagh BP (2006). The effect of global hypoxia on myocardial function after successful cardiopulmonary resuscitation in a laboratory model. Resuscitation.

[63] Toller W, Wolkart G, Stranz C, Metzler H, Brunner F (2005). Contractile action of levosimendan and epinephrine during acidosis. Eur J Pharmacol.

[64] Chan PS, Kereiakes DJ, Bartone C, Chow T (2008). Usefulness of microvolt T-wave alternans to predict outcomes in patients with ischemic cardiomyopathy beyond one year. Am J Cardiol.

[65] Rosenbaum DS, Jackson LE, Smith JM, Garan H, Ruskin JN, Cohen RJ (1994). Electrical alternans and vulnerability to ventricular arrhythmias. N Engl J Med.

[66] Austin C, Wray S (1993). Extracellular pH signals affect rat vascular tone by rapid transduction into intracellular pH changes. J Physiol.

[67] Austin C, Wray S (1993). Changes of intracellular pH in rat mesenteric vascular smooth muscle with high-K+ depolarization. J Physiol.

[68] Gardner JP, Diecke FP (1988). Influence of pH on isometric force development and relaxation in skinned vascular smooth muscle. Pflugers Arch.

[69] Mitchell JH, Wildenthal K, Johnson RL (1972). The effects of acid–base disturbances on cardiovascular and pulmonary function. Kidney Int.

[70] Fujita M, Asanuma H, Hirata A, Wakeno M, Takahama H, Sasaki H (2007). Prolonged transient acidosis during early reperfusion contributes to the cardioprotective effects of postconditioning. Am J Physiol Heart Circ Physiol.

[71] Steenbergen C, Deleeuw G, Rich T, Williamson JR (1977). Effects of acidosis and ischemia on contractility and intracellular pH of rat heart. Circ Res.

[72] Gabig TG, Bearman SI, Babior BM (1979). Effects of oxygen tension and pH on the respiratory burst of human neutrophils. Blood.

[73] Kin H, Zatta AJ, Lofye MT, Amerson BS, Halkos ME, Kerendi F (2005). Postconditioning reduces infarct size via adenosine receptor activation by endogenous adenosine. Cardiovasc Res.

[74] Yang XM, Proctor JB, Cui L, Krieg T, Downey JM, Cohen MV (2004). Multiple, brief coronary occlusions during early reperfusion protect rabbit hearts by targeting cell signaling pathways. J Am Coll Cardiol.

[75] Refsum HE, Opdahl H, Leraand S (1997). Effect of extreme metabolic acidosis on oxygen delivery capacity of the blood - an in vitro investigation of changes in the oxyhemoglobin dissociation curve in blood with pH values of approximately 6.30. Crit Care Med.

[76] Siegel G, Emden J, Wenzel K, Mironneau J, Stock G (1992). Potassium channel activation in vascular smooth muscle. Adv Exp Med Biol.

[77] Jennings RB, Reimer KA, Steenbergen C, Schaper J (1989). Total ischemia III: effect of inhibition of anaerobic glycolysis. J Mol Cell Cardiol.

[78] Neumar RW, Otto CW, Link MS, Kronick SL, Shuster M, Callaway CW (2010). Part 8: adult advanced cardiovascular life support. 2010 American Heart Association Guidelines for Cardiopulmonary Resuscitation and Emergency Cardiovascular Care. Circulation.

[79] Bollaert PE, Robin-Lherbier B, Mallie JP, Nace L, Escanye JM, Larcan A (1994). Effects of sodium bicarbonate on striated muscle metabolism and intracellular pH during endotoxic shock. Shock.

[80] Stacpoole PW (1986). Lactic acidosis: the case against bicarbonate therapy. Ann Intern Med.

[81] Wilson RF, Spencer AR, Tyburski JG, Dolman H, Zimmerman LH (2013). Bicarbonate therapy in severely acidotic trauma patients increases mortality. J Trauma Acute Care Surg.

[82] Arieff AI, Leach W, Park R, Lazarowitz VC (1982). Systemic effects of NaHCO3 in experimental lactic acidosis in dogs. Am J Physiol.

[83] Rhee KH, Toro LO, McDonald GG, Nunnally RL, Levin DL (1993). Carbicarb, sodium bicarbonate, and sodium chloride in hypoxic lactic acidosis. Effect on arterial blood gases, lactate concentrations, hemodynamic variables, and myocardial intracellular pH. Chest.

[84] Valenza F, Pizzocri M, Salice V, Chevallard G, Fossali T, Coppola S (2012). Sodium bicarbonate treatment during transient or sustained lactic acidemia in normoxic and normotensive rats. PLoS One.

[85] Boyd JH, Walley KR (2008). Is there a role for sodium bicarbonate in treating lactic acidosis from shock?. Curr Opin Crit Care.

[86] Lang RM, Fellner SK, Neumann A, Bushinsky DA, Borow KM (1988). Left ventricular contractility varies directly with blood ionized calcium. Ann Intern Med.

[87] Beech JS, Nolan KM, Iles RA, Cohen RD, Williams SC, Evans SJ (1994). The effects of sodium bicarbonate and a mixture of sodium bicarbonate and carbonate (“Carbicarb”) on skeletal muscle pH and hemodynamic status in rats with hypovolemic shock. Metabolism.

[88] Cooper DJ, Herbertson MJ, Werner HA, Walley KR (1993). Bicarbonate does not increase left ventricular contractility during L-lactic acidemia in pigs. Am Rev Respir Dis.

[89] Cooper DJ, Walley KR, Wiggs BR, Russell JA (1990). Bicarbonate does not improve hemodynamics in critically ill patients who have lactic acidosis. A prospective, controlled clinical study. Ann Intern Med.

[90] Graf H, Leach W, Arieff AI (1985). Evidence for a detrimental effect of bicarbonate therapy in hypoxic lactic acidosis. Science.

[91] Iberti TJ, Kelly KM, Gentili DR, Rosen M, Katz DP, Premus G, Benjamin E (1988). Effects of sodium bicarbonate in canine hemorrhagic shock. Crit Care Med.

[92] Mathieu D, Neviere R, Billard V, Fleyfel M, Wattel F (1991). Effects of bicarbonate therapy on hemodynamics and tissue oxygenation in patients with lactic acidosis: a prospective, controlled clinical study. Crit Care Med.

[93] Giunti C, Priouzeau F, Allemand D, Levraut J (2007). Effect of tris-hydroxymethyl aminomethane on intracellular pH depends on the extracellular non-bicarbonate buffering capacity. Transl Res.

[94] Moon PF, Gabor L, Gleed RD, Erb HN (1997). Acid–base, metabolic, and hemodynamic effects of sodium bicarbonate or tromethamine administration in anesthetized dogs with experimentally induced metabolic acidosis. Am J Vet Res.

[95] Sirieix D, Delayance S, Paris M, Massonnet-Castel S, Carpentier A, Baron JF (1997). Tris-hydroxymethyl aminomethane and sodium bicarbonate to buffer metabolic acidosis in an isolated heart model. Am J Respir Crit Care Med.

[96] Hoste EA, Colpaert K, Vanholder RC, Lameire NH, De Waele JJ, Blot SI, Colardyn FA (2005). Sodium bicarbonate versus THAM in ICU patients with mild metabolic acidosis. J Nephrol.

[97] Adrogue HJ, Madias NE (1998). Management of life-threatening acid–base disorders. First of two parts. N Engl J Med.

[98] Sonett J, Baker LS, Hsi C, Knox MA, Visner MS, Landow L (1993). Sodium bicarbonate versus Carbicarb in canine myocardial hypercarbic acidosis. J Crit Care.

[99] Bersin RM, Arieff AI (1988). Improved hemodynamic function during hypoxia with Carbicarb, a new agent for the management of acidosis. Circulation.

[100] Leung JM, Landow L, Franks M, Soja-Strzepa D, Heard SO, Arieff AI, Mangano DT (1994). Safety and efficacy of intravenous Carbicarb in patients undergoing surgery: comparison with sodium bicarbonate in the treatment of mild metabolic acidosis. SPI Research Group. Study of Perioperative Ischemia. Crit Care Med.

[101] Uchino S, Bellomo R, Ronco C (2001). Intermittent versus continuous renal replacement therapy in the ICU: impact on electrolyte and acid–base balance. Intensive Care Med.

[102] Tan HK, Uchino S, Bellomo R (2003). The acid–base effects of continuous hemofiltration with lactate or bicarbonate buffered replacement fluids. Int J Artificial Organs.

[103] Naka T, Bellomo R (2004). Bench-to-bedside review: treating acid–base abnormalities in the intensive care unit - the role of renal replacement therapy. Crit Care.

[104] Investigators RRTS, Bellomo R, Cass A, Cole L, Finfer S, Gallagher M (2009). Intensity of continuous renal-replacement therapy in critically ill patients. N Engl J Med.

[105] Bellomo R, Lipcsey M, Calzavacca P, Haase M, Haase-Fielitz A, Licari E (2013). Early acid–base and blood pressure effects of continuous renal replacement therapy intensity in patients with metabolic acidosis. Intensive Care Med.

[106] Cole L, Bellomo R, Journois D, Davenport P, Baldwin I, Tipping P (2001). High-volume haemofiltration in human septic shock. Intensive Care Med.

[107] Klouche K, Cavadore P, Portales P, Clot J, Canaud B, Beraud JJ (2002). Continuous veno-venous hemofiltration improves hemodynamics in septic shock with acute renal failure without modifying TNFalpha and IL6 plasma concentrations. J Nephrol.

[108] Ratanarat R, Brendolan A, Piccinni P, Dan M, Salvatori G, Ricci Z, Ronco C (2005). Pulse high-volume haemofiltration for treatment of severe sepsis: effects on hemodynamics and survival. Crit Care.

[109] Tani M, Neely JR (1990). Na + accumulation increases Ca2+ overload and impairs function in anoxic rat heart. J Mol Cell Cardiol.

[110] Wu D, Kraut JA, Abraham WM (2013). Sabiporide improves cardiovascular function, decreases the inflammatory response and reduces mortality in acute metabolic acidosis in pigs. PLoS One.

[111] Lin X, Lee D, Wu D (2014). Sabiporide improves cardiovascular function and attenuates organ injury from severe sepsis. J Surg Res.

[112] Kim HJ, Son YK, An WS (2013). Effect of sodium bicarbonate administration on mortality in patients with lactic acidosis: a retrospective analysis. PLoS One.

[113] Levraut J, Garcia P, Giunti C, Ichai C, Bouregba M, Ciebiera JP (2000). The increase in CO2 production induced by NaHCO3 depends on blood albumin and hemoglobin concentrations. Intensive Care Med.

[114] Nielsen HB, Bredmose PP, Stromstad M, Volianitis S, Quistorff B, Secher NH (2002). Bicarbonate attenuates arterial desaturation during maximal exercise in humans. J Appl Physiol (1985).

[115] Nakashima K, Yamashita T, Kashiwagi S, Nakayama N, Kitahara T, Ito H (1996). The effect of sodium bicarbonate on CBF and intracellular pH in man: stable Xe-CT and 31P-MRS. Acta Neurol Scand Suppl.

[116] Mark NH, Leung JM, Arieff AI, Mangano DT (1993). Safety of low-dose intraoperative bicarbonate therapy: a prospective, double-blind, randomized study. The Study of Perioperative Ischemia (SPI) Research Group. Crit Care Med.

[117] Fanconi S, Burger R, Ghelfi D, Uehlinger J, Arbenz U (1993). Hemodynamic effects of sodium bicarbonate in critically ill neonates. Intensive Care Med.

[118] Bersin RM, Chatterjee K, Arieff AI (1989). Metabolic and hemodynamic consequences of sodium bicarbonate administration in patients with heart disease. Am J Med.

[119] Shapiro JI, Whalen M, Chan L (1990). Hemodynamic and hepatic pH responses to sodium bicarbonate and Carbicarb during systemic acidosis. Magn Reson Med.

[120] Dimlich RV, Biros MH, Widman DW, Kaplan J (1988). Comparison of sodium bicarbonate with dichloroacetate treatment of hyperlactatemia and lactic acidosis in the ischemic rat. Resuscitation.

[121] Hope PL, Cady EB, Delpy DT, Ives NK, Gardiner RM, Reynolds EO (1988). Brain metabolism and intracellular pH during ischaemia: effects of systemic glucose and bicarbonate administration studied by 31P and 1H nuclear magnetic resonance spectroscopy in vivo in the lamb. J Neurochem.

[122] Sessler D, Mills P, Gregory G, Litt L, James T (1987). Effects of bicarbonate on arterial and brain intracellular pH in neonatal rabbits recovering from hypoxic lactic acidosis. J Pediatr.

[123] Graf H, Leach W, Arieff AI (1985). Metabolic effects of sodium bicarbonate in hypoxic lactic acidosis in dogs. Am J Physiol.

