# Erratum to: Hemodynamic consequences of severe lactic acidosis in shock states: from bench to bedside

**DOI:** 10.1186/s13054-017-1624-2

**Published:** 2017-02-21

**Authors:** Antoine Kimmoun, Emmanuel Novy, Thomas Auchet, Nicolas Ducrocq, Bruno Levy

**Affiliations:** 1 CHU Nancy, Service de Réanimation Médicale Brabois, Pole Cardiovasculaire et Réanimation Médicale, Hôpital de Brabois, Vandoeuvre-les-Nancy, 54511 France; 2 Université de Lorraine, Nancy, 54000 France; 3 INSERM U1116, Groupe Choc, Faculté de Médecine, Vandoeuvre-les-Nancy, 54511 France

## Erratum

Following publication of our article in *Critical Care* [1], the following error was brought to our attention. The sentence that reads “The rise in carbon dioxide partial pressure also increases hemoglobin affinity for oxygen and may, therefore, decrease oxygen delivery” is incorrect. The words “increases” and “decrease” were reversed.

The correct sentence should read: “The rise in carbon dioxide partial pressure also decreases hemoglobin affinity for oxygen and may, therefore, increase oxygen delivery.” The original article also unfortunately published with the incorrect cover date. This was published with a cover date December 2015 whereas this should have been January 2016. This has been updated.
